# Redefining Digital Dentistry: Multidisciplinary Applications of 3D Printing for Personalized Dental Care

**DOI:** 10.7759/cureus.86791

**Published:** 2025-06-26

**Authors:** Ruqayyah Almarshadi, Sabreen Hamdi, Fatimah Hadi, Aisha Alshehri, Reem Alsahafi, Nouf Aljohani, Rafan Alyamani, Mariam Alhbchi, Nadeen Alqahtani, Reem Almutiri, Thageba Alqadi, Afnan Alhabardi, Kawthar Abu Aziz, Talal Alshammeri

**Affiliations:** 1 College of Dentistry, University of Hail, Hail, SAU; 2 College of Dentistry, Jazan University, Jazan, SAU; 3 Faculty of Dentistry, King Abdulaziz University, Jeddah, SAU; 4 College of Dentistry, Majmaah University, Majmaah, SAU; 5 College of Dentistry, Riyadh Elm University, Riyadh, SAU; 6 College of Dentistry, Qassim University, Buraydah, SAU; 7 College of Dentistry, Imam Abdulrahman Bin Faisal University, Dammam, SAU; 8 Board of Oral and Maxillofacial Surgery, King Salman Specialist Hospital, Hail, SAU

**Keywords:** 3d printing, additive manufacturing, digital dentistry, digital workflow, technology

## Abstract

The integration of 3D printing into dentistry has led to a revolution in the precision and personalization of dental care. This review examines the extensive applications of 3D printing technology in various branches of dentistry. With the capability to create highly detailed, patient-specific models, devices, and appliances, 3D printing is transforming clinical workflows, enhancing the accuracy of treatments, and reducing procedural time. Moreover, it supports a digital workflow that aligns with the growing trend of personalized healthcare. Innovations in printer resolution, biocompatible materials, and printing speed continue to push the boundaries of what is possible in dental care. However, limitations related to material properties, regulatory considerations, and the need for specialized training persist. The review also highlights ongoing advancements in 3D printing technology and materials, which promise to further revolutionize dental practice. In conclusion, 3D printing holds immense potential for enhancing dental care, although overcoming existing challenges will require ongoing research and innovation.

## Introduction and background

Additive manufacturing, commonly known as 3D printing, has made substantial inroads into various fields of healthcare, with dentistry being one of the most rapidly advancing sectors. The technology enables the creation of highly detailed and accurate 3D objects directly from digital designs, significantly enhancing dental procedures. In the context of dentistry, 3D printing facilitates the production of dental implants, crowns, bridges, dentures, and orthodontic appliances, as well as surgical guides, all with unprecedented levels of precision and customization [1].

Historically, the process of creating dental restorations and devices has been labor-intensive, relying on manual techniques such as traditional impressions and casting. With the advent of 3D printing, these processes have undergone a revolution. Digital technologies, such as cone-beam computed tomography (CBCT) and intraoral scanning, have enabled the creation of digital representations of patients' oral structures [2]. These digital files can be fed into 3D printers to produce models and devices that match the patient's anatomy exactly, leading to improved clinical outcomes and a reduction in the time from diagnosis to treatment. Additionally, 3D printing offers significant cost savings by reducing the need for labor-intensive procedures and materials, thus making advanced dental care more accessible [3].

However, while the benefits of 3D printing are evident, challenges persist in its broader application. The availability of biocompatible materials that can withstand the rigors of long-term use in the oral environment remains a key barrier. Moreover, regulatory frameworks for 3D-printed dental products are still developing, which can cause delays in the adoption of this technology. This narrative review examines the diverse applications of 3D printing in dentistry, assesses its current limitations, and discusses future directions for the technology in this field.

## Review

Search strategy

A comprehensive literature search was conducted across major electronic databases, including PubMed, Scopus, and Web of Science. The search utilized a combination of keywords, including "3D printing," "additive manufacturing," and "digital dentistry." Reference lists of relevant articles were also screened to identify additional studies. Both original research articles and relevant reviews were considered to provide an overview of the applications, advantages, limitations, and future perspectives of 3D printing technology in dentistry. The selected literature was critically analyzed to summarize current knowledge and emerging trends in the field.

Oral and maxillofacial surgery

In the realm of oral and maxillofacial surgery (OMS), 3D printing has dramatically improved the accuracy and efficiency of both pre-surgical planning and actual surgical procedures. The ability to create patient-specific anatomical models from medical imaging data enables surgeons to visualize and rehearse complex procedures prior to performing them on the patient.

Surgical Planning Models

One of the most transformative uses of 3D printing in OMS is the fabrication of surgical planning models. These models are anatomically accurate representations of the patient's skull, jaw, or facial bones, derived from high-resolution imaging techniques like CBCT or CT scans [4].

These 3D models allow surgeons to study and plan complex surgical procedures in detail before entering the operating room. By providing a tangible, 3D representation of the patient's anatomy, surgical planning models enable better visualization of deformities, fractures, or anatomical anomalies. This enhanced understanding of the patient's condition can significantly improve surgical outcomes by allowing surgeons to rehearse procedures, anticipate challenges, and develop optimal surgical strategies [5].

Surgical planning models are especially valuable in trauma cases, where rapid decisions must be made regarding the repair of fractured bones, as well as in tumor resection surgeries, where the exact extent of bone removal must be carefully planned. They also play a crucial role in orthognathic surgery, which involves repositioning the jaw to correct skeletal discrepancies. Furthermore, these models are useful for patient education, allowing patients to better understand their condition and the planned surgery, which can help reduce anxiety and improve patient compliance [6].

Patient-Specific Implants

Patient-specific implants (PSIs) are custom-designed implants tailored to fit the precise anatomy of a patient's jaw or facial structure. These implants are typically made from biocompatible materials, such as titanium or polyether ether ketone, and are designed using 3D models created from the patient's imaging data. PSIs are particularly valuable in cases of orbital floor reconstruction, zygomatic bone fractures, and mandibular defects following tumor resection [7].

By using 3D printing to produce PSIs, surgeons can ensure a precise fit, which can be challenging with conventional, off-the-shelf implants. The custom design of PSIs reduces the need for extensive intraoperative adjustments, thereby minimizing surgical time and improving the overall efficiency of the procedure. This also leads to better functional outcomes, as the implant will more accurately replicate the natural anatomy, supporting the restoration of normal bone function and aesthetics [8]. Moreover, the use of PSIs reduces the risk of complications such as infection or implant failure, as these implants are designed to integrate seamlessly with the patient's anatomy. The ability to produce these implants on demand also reduces the time required for preoperative preparation, ensuring faster recovery times [9].

Cutting and Drilling Surgical Guides

Cutting and drilling guides are 3D-printed surgical tools designed to enhance the precision of various OMS procedures. These guides are particularly useful in complex surgeries, such as bone resection in tumor or cyst excision, osteotomies in orthognathic surgery, and implant placement in the jaw. These guides are designed to fit precisely on the patient's anatomical structures, providing exact templates for cutting and drilling during surgery. For example, in tumor resection surgeries, a 3D-printed guide can help ensure that the surgeon removes only the tumor while preserving as much healthy tissue as possible. Similarly, in orthognathic surgery, the guide ensures that bone cuts are made at the correct angles, which is critical for achieving optimal results [10]. The primary advantage of 3D-printed surgical guides is their ability to increase precision and minimize human error. In complex surgeries, even slight deviations from the planned incisions can lead to significant complications. Three-dimensional printing ensures that the guide fits the patient's unique anatomy, allowing for more predictable and successful surgical outcomes [11].

Mandibular and Maxillary Reconstruction

Mandibular and maxillary reconstructions are common in OMS, particularly after trauma, tumor resection, or congenital deformities. One of the key challenges in these procedures is the need for bone regeneration and the creation of stable, functional structures. Three-dimensional printing has enabled the production of scaffolds for bone regeneration using bioresorbable materials, which gradually integrate into the patient's tissue over time, promoting natural healing [12]. For complex reconstructions, 3D printing can also be used to create custom reconstruction plates that fit the patient's unique anatomy, including the jaw and facial bones. These plates are often used after trauma or surgery to provide support and facilitate healing. The ability to design and print these plates ensures that they are perfectly aligned with the patient's bone structure, improving both the functional and aesthetic outcomes of the reconstruction [13]. Furthermore, 3D printing facilitates guided bone regeneration (GBR), where scaffolds can be used to support the growth of new bone tissue in areas that have been severely damaged. These bioresorbable scaffolds provide a matrix that allows bone cells to grow and integrate, ultimately regenerating the lost or damaged bone tissue [14].

Temporomandibular Joint Prosthesis Design

In patients with temporomandibular joint (TMJ) disorders, including ankylosis, degenerative disease, or damage following oncological resection, 3D printing has enabled the creation of customized TMJ prostheses. These prostheses are designed to match the patient's specific anatomical requirements, ensuring proper function and comfort [15]. Using 3D-printed models, surgeons can design TMJ prostheses that replicate the natural joint structure, reducing the risk of complications and improving postoperative outcomes. Customization enables better alignment and articulation of the joint, which is crucial for restoring normal jaw movement and preventing further deterioration [16]. Moreover, 3D printing allows the production of TMJ prostheses with a high degree of precision, which can be challenging to achieve using traditional manufacturing methods. This leads to a more functional, durable, and comfortable prosthesis, which is crucial for patients who have undergone TMJ-related surgeries [17].

Orthognathic and Craniofacial Surgery

Orthognathic surgery involves the repositioning of the jaw to correct skeletal discrepancies and improve both function and appearance. Virtual surgical planning (VSP) integrated with 3D-printed guides has become an indispensable tool in this field. VSP enables surgeons to plan surgeries digitally by simulating various procedures, including Le Fort osteotomy, bilateral sagittal split osteotomy, genioplasty, and cranial vault surgery [18]. The combination of VSP and 3D-printed guides provides precise, preoperative simulation, allowing for optimal bone repositioning with minimal intraoperative adjustments. This approach enhances the accuracy and predictability of the surgery, leading to improved functional and aesthetic outcomes. Furthermore, the use of 3D-printed surgical guides helps minimize the risk of complications, such as nerve damage or jaw misalignment, which can occur if the surgery is performed without precise planning [19]. By incorporating 3D printing into orthognathic and craniofacial surgeries, surgeons can also perform more complex reconstructions, particularly in cases involving congenital defects or trauma. The technology has revolutionized how surgeons approach the correction of facial deformities, enhancing both the technical and cosmetic results.

Distraction Osteogenesis Devices

Distraction osteogenesis is a surgical technique used to gradually lengthen bones, often used in pediatric craniofacial surgery to correct congenital anomalies, such as craniofacial microsomia or cleft lip and palate. Three-dimensional printing plays a vital role in the development of custom distraction devices that can be tailored to the individual patient's anatomy. These devices, which help guide bone lengthening, can be printed with patient-specific dimensions, ensuring a precise fit and optimal functionality. The customization provided by 3D printing enhances the success of distraction osteogenesis by allowing for the gradual and controlled lengthening of bones, minimizing the risk of complications such as infection or misalignment [20]. Additionally, 3D printing accelerates the prototyping process of distraction devices, enabling quicker adaptation to a patient's changing anatomical needs as the procedure progresses. This leads to more predictable and effective outcomes, particularly in pediatric patients who may require frequent adjustments as they grow [21].

Tissue Engineering and Regeneration

An exciting frontier in OMS is the use of bioprinting for tissue engineering and regeneration. Three-dimensional bioprinting utilizes living cells, biomaterials, and growth factors to create scaffolds that stimulate the regeneration of both soft and hard tissues [22]. This technology holds great promise for the restoration of bone and cartilage, particularly in cases of severe trauma, congenital defects, or tumor resections. By printing cell-seeded constructs, surgeons can create patient-specific scaffolds that encourage the growth of new tissues, aiding in the healing and restoration of damaged or missing bone and cartilage [23]. The potential for personalized bone and cartilage restoration could revolutionize reconstructive surgery, offering more natural and functional solutions compared to traditional methods. While still in its early stages, tissue engineering and regeneration via 3D bioprinting are exciting areas of research with the potential to significantly enhance the success of oral and maxillofacial surgeries in the future.

Education and Simulation

**Figure 1:**
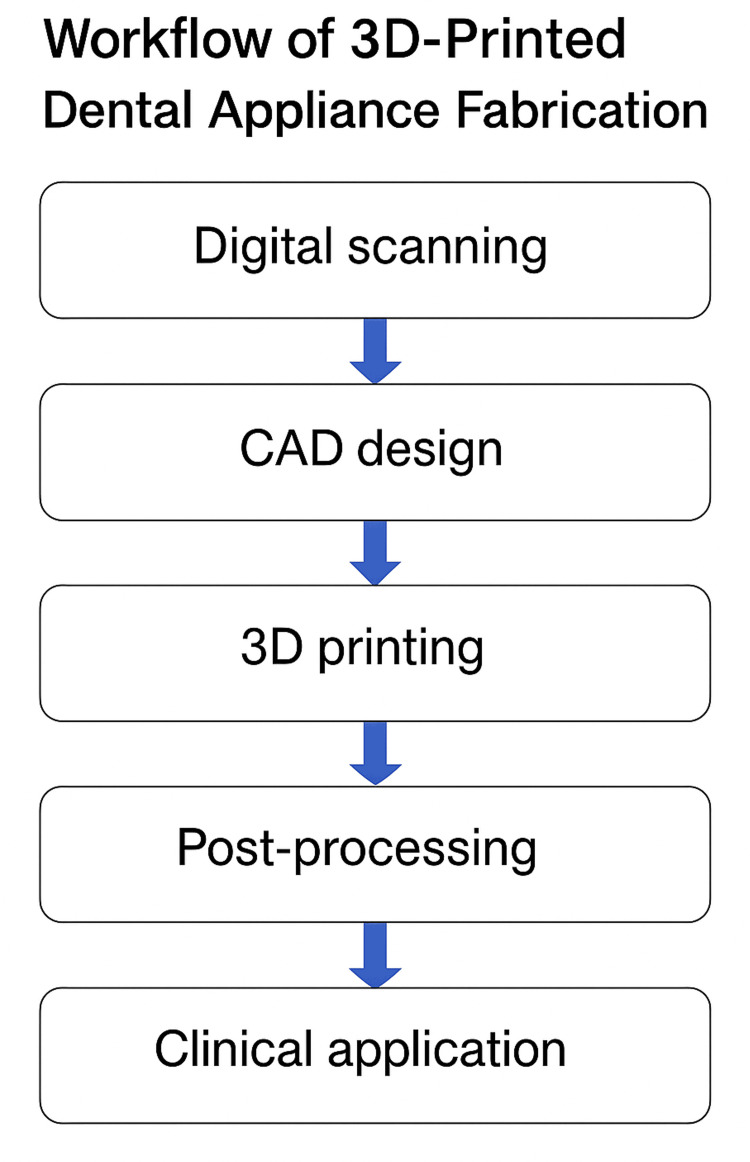
Stepwise process of additive manufacturing in dentistry 3D: three dimensional, CAD: computer-aided design Visualization was done with the help of Canva (www.canva.com).

Three-dimensional printing has proven to be an invaluable tool in education and simulation within the field of OMS. Realistic 3D-printed models of anatomical structures allow students and residents to practice and refine their surgical skills on lifelike simulations before performing actual surgeries. These models can be used to simulate a wide range of conditions, from common fractures to rare and complex surgeries. The use of 3D-printed training models enhances the educational experience by providing hands-on practice with accurate representations of human anatomy. Moreover, they enable the simulation of rare or complex surgeries, allowing trainees to gain exposure to challenging cases without the risk of patient harm. This approach accelerates learning and improves the competence of future oral and maxillofacial surgeons [24]. A schematic representation of the digital workflow involved in creating 3D-printed dental appliances is depicted in Figure 1.

Cleft patients

Cleft lip and palate are among the most common congenital deformities, often requiring comprehensive multidisciplinary care throughout a patient's life. The use of 3D printing technology in cleft care has revolutionized treatment protocols, providing more accurate, efficient, and patient-specific solutions. With its ability to create custom, anatomically tailored devices and models, 3D printing significantly enhances presurgical planning, postoperative recovery, and overall patient comfort [25].

Nasoalveolar Molding Devices

One of the most notable innovations for cleft patients, particularly for those born with a cleft lip and palate, is the development of nasoalveolar molding (NAM) devices. NAM is a presurgical orthodontic technique used to align the alveolar segments and improve the shape of the nasal cavity in infants with cleft lip and palate. The goal of NAM is to reduce the severity of the cleft, enhance the position of the upper lip and alveolar ridge, and facilitate optimal surgical outcomes [26]. Traditionally, NAM devices were custom-made using manual techniques, which were time-consuming and often less precise. However, with the advent of 3D printing, NAM devices can now be produced much faster and with a higher degree of accuracy. The 3D-printed NAM appliances are tailored to the infant's unique anatomical needs based on digital impressions or scans, ensuring a precise fit. This precision significantly enhances the device's effectiveness in realigning the alveolar segments and shaping the nasal structures prior to surgery [27]. Another significant advantage of 3D printing for NAM devices is the digital NAM workflow, which allows for remote planning. This approach minimizes the need for multiple in-person visits, reducing travel burden for families and allowing for more consistent treatment, especially in remote or underserved areas. Additionally, the faster fabrication process ensures that NAM devices can be delivered promptly, which is critical in the early stages of treatment to achieve optimal results [28].

Palatal Obturators

For newborns with cleft palates, feeding can be a significant challenge due to the inability to create a proper seal for suction during breastfeeding. Palatal obturators and feeding plates are custom devices that can be used to help newborns feed more effectively prior to surgical repair of the cleft. These devices are designed to close the gap in the palate, making it easier for the infant to create suction and feed more efficiently, while also reducing the risk of aspiration and improving overall nutritional intake [29]. Three-dimensional printing offers significant advantages in making these devices, as they can be customized to the infant's unique anatomy based on digital scans or impressions. Traditional methods for fabricating palatal obturators involved the use of conventional impressions, which can be challenging to obtain in fragile newborns. Three-dimensional printing eliminates the need for physical impressions. It enables a faster fabrication process, ensuring that devices are ready for use more quickly, which is crucial for infants' nutrition and development. Moreover, 3D-printed palatal obturators can provide a more precise fit, enhancing comfort and effectiveness. The ability to precisely replicate the infant's anatomical structures enables these devices to restore function better and improve the patient's ability to feed. As with NAM devices, the digital workflows involved in the design and fabrication of palatal obturators allow for greater flexibility in remote consultations, reducing the need for multiple clinic visits and making it easier for families to access care [30].

Bone Graft Scaffolds and Reconstruction Plates

In cleft lip and palate cases, bone grafting is often necessary to repair the alveolar ridge and prepare the area for future dental implants. Traditionally, bone grafts were sourced from the patient's bone, or synthetic graft materials were used. However, with the development of 3D printing, patient-specific bone graft scaffolds and reconstruction plates can now be created to promote GBR, particularly in the area of the alveolar ridge. Patient-specific scaffolds are designed based on the patient's unique anatomical needs, allowing for a more precise fit. These scaffolds can be made from bioresorbable materials or titanium, depending on the specific requirements of the case. Bioresorbable scaffolds are particularly advantageous in that they gradually integrate with the patient's bone over time, supporting natural bone growth and eliminating the need for a second surgery to remove the scaffold. In contrast, titanium scaffolds provide more permanent support for the reconstruction, particularly in cases of extensive bone loss [31].

The use of 3D printing for bone graft scaffolds significantly improves the fit and adaptation of the grafts to the infant's palate. As newborns are particularly challenging to work with due to their small and fragile anatomy, 3D printing enables the fabrication of scaffolds with greater precision, eliminating the need for conventional impression-taking, which can be difficult or even traumatic for the infant [32].

The use of custom reconstruction plates in cleft surgery enables the stabilization of the alveolar ridge and surrounding bones, thereby facilitating better healing and recovery. These plates can be tailored to fit the specific contours of the patient's mouth, which is essential for successful bone grafting and eventual implant placement. The ability to print custom plates that are precisely designed for each patient helps reduce surgical time and minimizes complications related to poorly fitting devices. For cleft patients, particularly those requiring pre-implant bone reconstruction, these 3D-printed scaffolds and reconstruction plates offer better support for bone regeneration, ensuring a smoother recovery process and more predictable results. By improving the precision and speed of the reconstruction process, these innovations significantly enhance the overall treatment outcomes for patients with cleft lip and palate [33].

Implantology

The field of implantology has greatly benefited from the advent of 3D printing technology. One of the most prominent applications of 3D printing in implantology is the creation of customized surgical guides that enhance the precision of implant placement. Using digital impressions and radiographic imaging data, 3D printing enables the creation of surgical guides that are tailored to the patient's unique anatomy. These guides provide a roadmap for implant placement, ensuring that the implants are positioned with the highest degree of accuracy [34].

Custom 3D-printed guides enable a more predictable outcome in dental implant surgery, thereby reducing the likelihood of errors during the placement process. As a result, the healing time is often reduced, and the risk of complications such as implant failure is minimized. Moreover, the use of patient-specific guides enhances the aesthetic outcomes of implant placement by ensuring that the implants are positioned in alignment with the patient's natural dentition. Beyond surgical guides, 3D printing is also utilized in the creation of custom implant components, including abutments and crowns. These components can be designed to fit the precise dimensions of the implant and the surrounding tissues, resulting in a better fit and improved long-term outcomes. As the technology continues to evolve, the materials used in 3D-printed implants are also improving, with advances in biocompatible metals, ceramics, and polymers that offer enhanced durability and integration with bone [35].

Orthodontics

In orthodontics, 3D printing has revolutionized the production of custom orthodontic appliances, such as aligners, retainers, and brackets. Traditional methods of creating orthodontic devices typically require multiple impressions and manual labor. This enables orthodontists to develop exact appliances tailored to the individual needs of each patient.

Clear Aligners

One of the most widespread uses of 3D printing in orthodontics today is the production of clear aligners. Aligners are clear plastic trays used to gradually move teeth into their desired positions [36]. The process begins with a digital scan of the patient's teeth, followed by the creation of a digital setup that maps out the desired tooth movement. From this digital model, 3D-printed models are generated in a sequence, each one representing a different stage of the treatment [37]. The key advantage of using 3D printing for aligners is that it enables customized treatment planning. Each aligner is explicitly designed to fit the patient's teeth and progressively move them into the correct position. This customization allows orthodontists to plan and execute more precise and efficient treatments, optimizing the tooth movement process for each patient [38].

The use of thermoformed aligners has significantly improved the turnaround time for creating these devices compared to 3D-printed models. Unlike traditional methods, which require multiple visits to take impressions and make molds, 3D printing enables fast production and remote consultations, as digital models can be shared and adjusted virtually, thereby reducing the need for physical appointments. Additionally, the ability to create multiple sequential models in a single process ensures that each aligner fits with great accuracy, further enhancing treatment efficiency and patient comfort [39].

Custom Brackets and Indirect Bonding Trays

Three-dimensional printing has also greatly impacted the creation of custom brackets and indirect bonding trays in orthodontics. Traditionally, brackets were manufactured as one-size-fits-all or in standardized designs; however, 3D printing enables the creation of custom base brackets tailored to each tooth's unique surface. The use of custom brackets offers several benefits. First, it allows for better precision in fitting, as the brackets are specifically designed to align with the individual's tooth anatomy. This customization leads to more efficient tooth movement and can reduce the treatment time required [40]. Additionally, indirect bonding trays can be created using 3D printing to ensure the precise placement of each bracket on the tooth. These trays ensure that the brackets are placed with great accuracy, reducing the risk of errors and the need for readjustments. The use of 3D printing for creating brackets and bonding trays reduces chairside time, as the orthodontist does not need to manually place each bracket, and it minimizes the need for patient adjustments. This leads to a more comfortable and faster treatment process for the patient while also increasing the overall efficiency of the orthodontic practice [41].

Removable Appliances and Functional Devices

Orthodontists also rely on removable appliances and functional devices to aid in the correction of dental and skeletal malocclusions. These devices are commonly used for minor corrections, retention after orthodontic treatment, and the correction of jaw discrepancies. With 3D printing, these appliances can now be digitally designed and printed directly or indirectly, offering several advantages over traditional methods. Three-dimensional printing enables the creation of appliances specifically designed to fit the patient's mouth, ensuring both effectiveness and comfort [42].

For instance, Hawley retainers, commonly used after orthodontic treatment to maintain tooth position, can now be quickly designed and printed to the exact specifications of the patient's teeth, providing better comfort and a more precise fit. Similarly, twin blocks used for correcting jaw discrepancies can be created with greater accuracy, ensuring more effective correction. The ability to produce these devices through 3D printing enhances the quality and efficiency of orthodontic care, resulting in improved patient satisfaction [43].

Fixed Appliances and Bands

With 3D printing, components like molar bands, lingual arch components, and habit-breaking appliances can be customized based on intraoral scans, making the process more efficient and accurate. One of the key benefits of 3D printing in the production of fixed appliances is the ability to tailor the devices to the specific anatomy of the patient's teeth. For example, molar bands can be 3D-printed with precise measurements, eliminating the need for off-the-shelf options that may require adjustments during the insertion process. By customizing these components to the patient's unique anatomy, the fit is significantly improved, reducing discomfort and increasing the effectiveness of the appliance [44].

Additionally, habit-breaking appliances, such as those used to help stop thumb-sucking or other undesirable habits, can be created using 3D printing to ensure a precise fit, making them more comfortable and effective. The ability to customize and digitally design these appliances allows orthodontists to provide patients with better care, reducing treatment time and improving long-term outcomes [45].

Study Models and Digital Documentation

Study models are used in orthodontics for diagnosis, treatment planning, and progress evaluation. Traditionally, study models were created from plaster impressions, which required significant storage space and could be prone to distortion over time. However, with 3D printing, orthodontists can now produce high-resolution digital study models that replace traditional plaster models, offering several advantages. Digital study models can be stored digitally, eliminating the need for physical space and reducing the risk of model degradation over time. Furthermore, the high resolution of 3D-printed models ensures that they accurately represent the patient's teeth, which is essential for diagnosis and treatment planning. The ability to evaluate a patient's progress using digital models also improves the precision of treatment adjustments and provides more accurate tracking over time. These digital models can be easily shared with other healthcare providers or specialists, enhancing collaboration in multidisciplinary treatment plans. Additionally, digital models can be used to generate various orthodontic devices, such as aligners or retainers, directly from the model, streamlining the workflow and reducing overall treatment time [46].

Surgical Guides in Orthodontics

Surgical procedures in orthodontics, such as the placement of temporary anchorage devices (TADs) or corticotomies, often require high precision to ensure safe and effective outcomes. Three-dimensional printing enables the creation of custom surgical guides that accurately and safely guide the placement of these devices. For example, TAD placement involves anchoring devices to the bone to help move teeth into their correct position. The placement of these devices requires precise positioning, and a 3D-printed surgical guide can be used to ensure that the TAD is placed in the correct location [47].

Similarly, corticotomies or piezocision-assisted orthodontics, which are minimally invasive surgical techniques to accelerate tooth movement, also benefit from 3D-printed surgical guides. These guides help ensure that the bone is cut in the correct areas and to the correct depth, thereby increasing the effectiveness of the procedure. By using 3D printing to create custom surgical guides, orthodontists can ensure a higher degree of precision and reduce the risk of complications, leading to better overall outcomes for patients undergoing surgical orthodontic procedures [48].

Retainers

Traditionally, retainers were fabricated from molds taken of the patient's teeth; however, with the rise of 3D printing, directly 3D-printed retainers are becoming increasingly common. These retainers, often made from clear trays, can be produced more quickly and efficiently using 3D printing. The benefit of 3D printing is that the retainers can be made from high-precision digital models of the patient's teeth, ensuring a perfect fit and greater comfort. Additionally, hybrid materials that combine both flexibility and strength are being developed, offering better long-term performance. Although 3D-printed retainers are still under development in some cases, they hold significant promise for reducing the fabrication steps, improving patient comfort, and ensuring that the retainer fits exactly as needed for effective retention after orthodontic treatment [49].

Pediatric dentistry

In pediatric dentistry, 3D printing has rapidly gained importance due to its ability to streamline treatment for children and alleviate many of the challenges associated with their care. Pediatric patients often have limited patience, strong gag reflexes, and heightened anxiety during dental visits. By utilizing digital workflows and additive manufacturing, clinicians can reduce chairside time and the number of appointments required, a crucial benefit for managing children's behavior [50]. The precision and customization offered by 3D printing also mean that devices can be made to fit a child's unique anatomy, improving comfort and outcomes.

Customized Orthodontic Appliances for Children

Three-dimensional printing has expanded the possibilities for pediatric orthodontic and orthopedic appliances beyond what traditional methods allow. Removable functional appliances can be custom-fitted through digital design, providing a precise fit that enhances wearability. Early reports suggest that patient-specific 3D-printed functional appliances achieve better intraoral fit, which may translate to higher acceptance and compliance in young patients​ [51]. In essence, clinicians can design an appliance on a computer using an intraoral scan and then fabricate it in-house with exact dimensions. This level of customization is especially valuable for children with mixed dentitions or unique oral anatomies, where one-size-fits-all solutions often fall short. Additionally, the digital workflow can incorporate growth predictions or virtual treatment planning, allowing clinicians to visualize facial changes and adjust appliance designs accordingly for their growing patients. The result is a more efficient and child-friendly approach to orthodontic care: appliances that fit better, work more effectively, and can often be produced faster than with conventional lab fabrication. Notably, 3D printing even opens the door to creative personalization. Devices could be printed with child-friendly designs, potentially reducing the intimidation factor for pediatric patients​ [52].

3D-Printed Space Maintainers

Space maintainers are crucial in pediatric dentistry for preserving arch length after the premature loss of primary teeth; however, conventional fabrication is labor-intensive and challenging for children who are uncooperative. A traditional band-and-loop space maintainer typically requires multiple appointments and technical steps, including fitting a metal band, taking an impression with the band in place, pouring a model, soldering a wire loop to the band on the model, and then cementing the appliance during a subsequent visit. This lengthy process not only tests a child's patience but also introduces many points where errors or misfits can occur [53]. With 3D printing, this process can be dramatically simplified. In a fully digital workflow, the dentist can take an intraoral scan of the child's teeth and then design a precise band-and-loop appliance digitally. The appliance can be 3D-printed in one piece and delivered in the same visit or shortly thereafter [54]. By fabricating the maintainer as a single, customized unit, the need for soldering is eliminated, and the fit to the tooth can be exact, thereby reducing the risk of decementation or failure [55]. This is especially advantageous for children with special needs or limited ability to cooperate, describing using a digital impression and 3D printer to make a band-and-loop maintainer in one session for children with ADHD, avoiding the "time and tedious workflow" of the conventional technique. Early clinical evidence indicated that 3D-printed space maintainers perform as well as or better than traditional ones [56]. From a patient's perspective, the custom fit and often smoother, monolithic design of a printed space maintainer can be more comfortable and easier to keep clean. There is also an aesthetic benefit: printed retainers can be made with tooth-colored coating or composite additions, making them far less visible than shiny metal loops​ [54]. Overall, the advent of 3D printing in space maintenance has transformed it into a quicker, one-visit procedure with appliances that are more durable and precisely fitted to the child's dentition, thereby improving both efficacy and the child's experience.

Pediatric Restorative and Prosthetic Devices

Additive manufacturing is also making inroads in restoring decayed or missing teeth in children. One notable application is the fabrication of custom pediatric crowns. Traditionally, when a baby tooth requires full coverage, clinicians rely on prefabricated stainless steel crowns or stock ceramic crowns that come in a limited range of sizes. These often require significant chairside adaptation and may not perfectly match the tooth's anatomy or the child's aesthetic needs. With 3D printing, clinicians can design a crown to the exact shape and dimensions of the prepared tooth, yielding a restoration that fits more precisely and looks more natural. The process typically involves scanning the tooth preparation and either printing the crown directly in a biocompatible material or printing a mold used to form the crown [52]. The speed of 3D printing is also beneficial in pediatric restorative situations; a custom crown or appliance can be produced rapidly, which is helpful in emergencies or to shorten chair time for anxious children. Beyond crowns, 3D printing can produce other prosthetic aids for children. In cases where a child has lost multiple teeth (due to trauma, caries, or developmental conditions), removable pediatric partial dentures or functional prostheses can be 3D printed to restore function and aesthetics. These appliances, sometimes required in young patients with congenitally missing teeth or after oral surgery, can be made thinner and lighter using 3D printing while still fitting securely, thereby improving the child's comfort compared to bulkier traditional acrylic appliances [57].

Another emerging use is in pediatric endodontics and surgery. Three-dimensional-printed surgical guides can assist in precisely locating and accessing calcified root canals in young permanent teeth or in guiding implant placement in growing jawbones when implants are indicated in adolescence [58]. While such cases are specialized, they underscore the versatility of 3D printing across various pediatric dental procedures. Importantly, all these restorative applications stand to benefit children by reducing invasive procedures and by producing restorations and devices that are customized to the child, often yielding superior fit and aesthetics compared to off-the-shelf solutions.

Educational Models and Training Aids

Three-dimensional printing's impact in pediatric dentistry extends beyond direct treatment devices; it is also a powerful tool for education and training. Dental educators and pediatric specialists can create highly accurate 3D-printed models of pediatric oral anatomy, which serve as invaluable simulators for teaching and rehearsal. For instance, a dental student or resident might practice a pulpotomy or stainless steel crown prep on a 3D-printed model of a child's tooth that mimics the real size and internal structure (including pulp horns) of a primary tooth. Complex anatomical scenarios can be replicated in resin models to help clinicians visualize and plan procedures before treating the actual child. Studies have shown that 3D-printed teaching models, complete with realistic tooth and gum textures, enhance students' understanding of pediatric dental anatomy and procedures, enabling them to navigate treatments virtually and gain confidence​ [59].

In practice, having a printed replica of a child patient's dentition or a specific pathology can help the dental team analyze the case in detail and strategize the best approach, which is particularly useful for complicated cases. Beyond training professionals, 3D-printed models play a significant role in patient and parent education. Dentists can use a physical 3D model of a child's teeth to explain treatment needs tangibly, for example, by showing how a cavity will be fixed or how an orthodontic appliance will create space for erupting teeth. This visual and hands-on educational approach makes it easier for children (and their parents) to grasp abstract dental concepts. Indeed, seeing a model of their mouth or the device that will be used can help demystify the procedure for a child and reduce their fear of it.

Restorative and prosthetic dentistry

In restorative and prosthetic dentistry, 3D printing has enhanced both the precision and speed of creating crowns, bridges, dentures, and other dental restorations. Traditionally, the creation of these devices required multiple patient visits and time-consuming manual labor. With 3D printing, however, digital impressions can be used to design and print highly accurate dental restorations that fit the patient's oral anatomy with minimal adjustment.

Crowns, Bridges, and Veneers

Crowns fabricated using 3D printing technology are highly accurate, as the digital model used to produce the crown is based on an intraoral scan of the patient's tooth. This results in a more comfortable fit and reduced need for adjustments. The use of 3D printing enables the creation of crowns from various materials, including ceramics, zirconia, and composite resins, all of which can replicate the natural appearance of teeth. Additionally, 3D printing allows for the production of crowns in a single visit, eliminating the need for temporary crowns or multiple appointments [60].

Bridges are used to replace one or more missing teeth by connecting a prosthetic tooth (pontic) to adjacent healthy teeth. Traditional bridgework requires meticulous preparation of abutment teeth, followed by multiple visits to complete the procedure. With 3D printing, however, entire bridges can be designed and produced from a digital scan in a much shorter time frame. The precision offered by 3D printing ensures that the bridge fits perfectly, reducing the need for adjustments. Furthermore, customized bridgework made using 3D printing provides better esthetics and functionality, as the pontic is designed to match the contours of the patient's mouth seamlessly [61].

Veneers are thin shells of tooth-colored material, typically made from porcelain or resin, that cover the front surface of teeth to improve their appearance. Veneers are primarily used for cosmetic purposes, such as enhancing the color, shape, or alignment of teeth. Three-dimensional printing enables the creation of highly accurate veneers that fit perfectly over the tooth, offering a more natural and durable solution compared to traditional veneer fabrication methods. Additionally, 3D printing allows for the production of veneers with a high degree of translucency, making them more lifelike and indistinguishable from natural teeth. The reduced chairside time and improved precision with 3D printing also result in better patient satisfaction [62].

Inlays, Onlays, and Overlays

Three-dimensional printing enables the precise fabrication of inlays based on a digital model of the tooth, resulting in a better fit and a more aesthetically pleasing restoration. Traditional methods often require multiple appointments to create inlays, but with 3D printing, inlays can be fabricated in a single visit, significantly reducing treatment time. Onlays are frequently used when there is more extensive damage or decay, but a full crown is not necessary. Similar to inlays, 3D printing has improved the precision of onlay restorations. Three-dimensional printers can create onlays with exacting precision, ensuring they fit well and integrate seamlessly with the surrounding tooth structure. This technology also enables the customization of onlays to match the patient's dental anatomy precisely, thereby improving both the functionality and aesthetic appeal of the restoration [63]. Overlays restore both the functionality and strength of the tooth, providing a more durable solution for teeth that have suffered substantial wear or damage. The precision afforded by 3D printing ensures that overlays can be designed to fit accurately and snugly onto the tooth, providing a more natural and functional bite. As with other restorative procedures, 3D printing reduces the need for multiple visits, leading to a quicker and more efficient restoration process [64].

**Figure 2:**
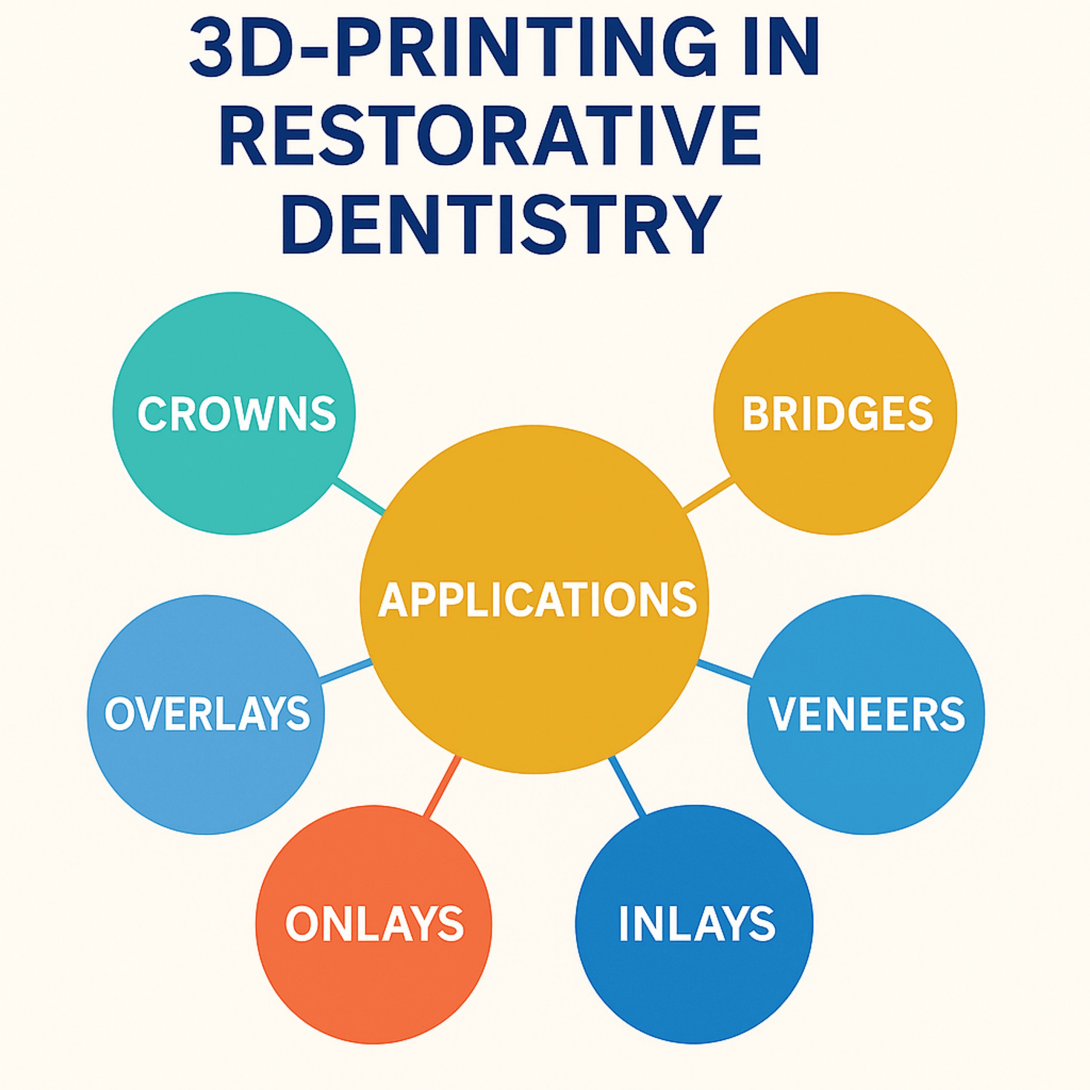
Schematic overview of 3D printing applications in restorative dentistry 3D: three dimensional Visualization was done with the help of Canva (www.canva.com).

The use of 3D printing for inlays, onlays, and overlays provides several advantages over traditional methods. The reduction in the number of required office visits, the improved fit of the restorations, and the ability to use high-quality materials such as porcelain or resin composites all contribute to better outcomes for patients. Additionally, the accuracy and precision of 3D printing help ensure that the restorations integrate well with the tooth surface, maintaining optimal function and esthetics [65]. Figure 2 illustrates the range of applications of 3D printing in restorative dentistry.

Endodontics

Endodontics has also benefited from the precision and customization offered by 3D printing. One of the most valuable applications of 3D printing in endodontics is the creation of custom root canal guides. These guides, based on 3D scans of the patient's teeth, ensure that the root canal system is accessed precisely, reducing the risk of damaging surrounding tissues and improving the overall efficiency of the procedure. Additionally, 3D printing allows for the creation of highly detailed models of the root canal system, which can aid in diagnosing and planning treatment for complex cases. The ability to visualize the root canal system in three dimensions enhances the clinician's understanding of the tooth's anatomy and improves the accuracy of cleaning and shaping the canals [66]. Post and core restorations are used to restore a tooth that has undergone root canal therapy. When a tooth's pulp is removed during a root canal procedure, the tooth may become weakened and require further reinforcement to support a crown. The post is placed inside the root canal to provide structural support, while the core is built up around the post to create a stable foundation for the final crown. Traditionally, post and core restorations have been created using prefabricated posts, which are then adapted to the shape of the root canal. However, 3D printing technology has significantly improved the process by enabling the creation of customized posts and cores specifically designed to fit the individual's root canal anatomy [67]. Using digital scanning and computer-aided design (CAD) software, dental professionals can create a post that fits perfectly within the canal, reducing the need for adjustments and improving the overall fit. The main advantage of 3D-printed post and core restorations is the high degree of accuracy in adapting the post to the root canal's shape and size. This custom fit significantly improves the stability of the post, ensuring that it effectively supports the final crown. Furthermore, 3D printing enables the use of materials that mimic the natural tooth structure, such as biocompatible resins or zirconia, thereby enhancing the strength and durability of the restoration [68].

Another benefit of 3D printing in post and core restorations is the ability to produce them more quickly. Traditional methods require multiple steps and office visits to take impressions, fabricate, and place the post and core. With 3D printing, the process can be completed in a single appointment, thereby improving the patient experience by reducing chairside time and accelerating the overall restoration process. The enhanced accuracy and efficiency of 3D printing in creating post-and-core restorations have made it an invaluable tool in modern endodontics. By providing a better-fitting post and core, this technology improves the long-term success and longevity of root canal-treated teeth, offering patients a more durable and functional solution [69]. Table 1 presents the key applications of 3D printing, summarizing the associated benefits and challenges.

**Table 1:** Applications of 3D printing across dental specialties 3D: three dimensional, PSIs: patient-specific implants, TMJ: temporomandibular joint, CAD: computer-aided design, NAM: nasoalveolar molding

Specialty	Key applications	Benefits	Challenges
Oral and maxillofacial Surgery	Surgical planning models, PSIs, cutting guides, TMJ prostheses, craniofacial reconstructions	Improved surgical accuracy, reduced operative time, personalized reconstructions	High cost of PSIs and custom devices, need for advanced imaging
Orthodontics	Clear aligners, custom brackets, bonding trays, study models	Faster appliance fabrication, better fit, improved patient comfort	Requires CAD expertise, material limitations for durability
Implantology	Surgical guides, custom abutments, implant planning models	Enhanced implant placement accuracy, reduced surgical complications	Cost of surgical guide production, regulatory approval hurdles
Pediatric dentistry	Space maintainers, NAM devices, custom crowns, educational models	Improved patient cooperation, faster treatment workflows, better fit for children	Cooperation during digital scanning, growth-related device adjustments
Restorative and prosthetic dentistry	Crowns, bridges, dentures, veneers, inlays/onlays/overlays	Faster turnaround time, superior precision, aesthetic outcomes	Material longevity concerns, initial cost of setup
Endodontics	Root canal guides, customized post-and-core systems	Increased procedural accuracy, reduced treatment time	Limited material choices for long-term intraoral use

Printing Technology in Dentistry

The success of 3D printing in dentistry is mainly due to the variety of technologies that enable high-precision manufacturing. Some of the most commonly used technologies in dental 3D printing include stereolithography apparatus (SLA), digital light processing (DLP), and fused deposition modeling (FDM). Each of these methods offers distinct advantages, depending on the desired application. SLA and DLP are particularly valued for their ability to produce highly detailed and precise objects with smooth surfaces, making them ideal for creating dental models, crowns, bridges, and surgical guides. SLA uses a laser to cure liquid resin layer by layer, while DLP uses a digital light projector to cure resin. Both technologies produce objects with high resolution, which is critical for dental applications where accuracy is paramount [70]. FDM, on the other hand, is a more widely used method for larger components, such as dentures and orthodontic appliances. Although it typically offers lower resolution compared to SLA and DLP, FDM is more affordable and efficient for producing larger quantities of parts, making it an attractive option for practices that require high-volume production of dental devices [71]. As 3D printing technologies continue to evolve, new advancements in both the printers themselves and the materials used in dental printing are expected to enhance the capabilities of 3D printing in dentistry. The development of new biocompatible materials, such as 3D-printable metals and ceramics, will enable the creation of stronger, more durable dental implants, crowns, and other restorations. Moreover, the integration of 3D printing with digital workflows, including intraoral scanning and CAD software, will streamline the process of creating dental restorations, making it faster and more cost-effective [72]. Table 2 provides a comparative overview of the advantages and disadvantages associated with the major 3D printing technologies utilized in dental practice.

**Table 2:** Comparison of advantages and disadvantages of 3D printing technologies used in dentistry 3D: three dimensional, SLA: stereolithography apparatus, DLP: digital light processing, FDM: fused deposition modeling, SLS: selective laser sintering

Printer type	Advantages	Disadvantages
SLA	Very high accuracy and fine detail, smooth surface finish, good for small, intricate parts, wide variety of dental-specific resins available	Brittle materials (especially older resins), requires careful post-curing, resins can be costly, messy post-processing workflow
DLP	Faster printing than SLA, high resolution, efficient for batch production, good mechanical properties in newer resins	Limited build volume (depending on printer), needs post-curing, resins can discolor over time, sensitivity to ambient light during printing
FDM	Very low cost (both printer and material), easy maintenance, fast prototyping, wide range of inexpensive materials	Poor surface finish (rougher texture), low resolution and detail, not suitable for final restorations, weak mechanical properties
SLS	Very strong, durable parts (especially nylon or metal), no need for support structures, suitable for complex geometries	Very high machine cost, surface finish often rough, expensive maintenance, requires specialized facilities (ventilation, powder handling)
PolyJet printing	Ultra-high resolution and detail, ability to combine different materials and colors, smooth, realistic models, ideal for soft tissue simulation	Very high cost for printer and materials, requires controlled environment, printed models can be sensitive to moisture, higher material waste

Limitations and future perspectives

Despite the clear benefits, several limitations of 3D printing in dentistry must be addressed. One of the primary challenges is the limited selection of materials currently available for 3D printing. Resins and plastics are commonly used. Yet, these materials may not always provide the same durability and long-term performance as traditional dental materials, such as porcelain, gold, or titanium. As a result, research into new, more durable, and biocompatible materials is critical for advancing the technology. Regulatory concerns also present a barrier to the widespread adoption of 3D printing in dentistry. Given that 3D-printed dental devices are often considered custom medical devices, they must undergo rigorous testing and regulatory approval before they can be used in clinical practice. Regulatory bodies, such as the FDA, are still working to establish clear guidelines for the approval of 3D-printed dental products, which can delay their availability and use. Additionally, the adoption of 3D printing requires significant investment in equipment and training. Dental professionals must become proficient in digital workflows, including scanning, designing, and printing dental devices, which requires time and resources. However, as the technology becomes more affordable and user-friendly, it is likely that more dental practitioners will incorporate 3D printing into their practices. Nevertheless, the future of 3D printing in dentistry appears promising. With ongoing research into advanced materials, software, and printing technologies, 3D printing is expected to continue improving the accuracy, customization, and cost-effectiveness of dental treatments. The widespread adoption of 3D printing is likely to lead to a more streamlined, patient-centric approach to dental care, with the potential for faster treatment times, improved outcomes, and reduced costs.

## Conclusions

Three-dimensional printing technology has become an integral tool in modern dentistry, offering unprecedented levels of precision, customization, and efficiency across various dental disciplines. By enabling the creation of patient-specific models, devices, and implants, 3D printing has the potential to significantly improve patient care and treatment outcomes. However, challenges related to material limitations, regulatory issues, and the need for specialized training must be addressed before the technology can be fully integrated into routine dental practice. As research and innovation in 3D printing continue, the future of this technology holds tremendous promise for advancing dental care.
